# Assessment of Caregiver Burden and Perceived Social Support Among Individuals Providing Care to Patients Under Home Health Services: A Descriptive Study

**DOI:** 10.1111/scs.70285

**Published:** 2026-07-01

**Authors:** Fatih Oğur, Mehmet Sait Değer, Kübra Temel Aslan, Hamza Akay, Abdülaziz İşılak, Bilal Baykal, Kübra Ayan, Elif Alaybay, Dilara Gür, Zeynep Meva Altaş

**Affiliations:** ^1^ İdil State Hospital Şırnak Türkiye; ^2^ Cerrahpaşa Faculty of Medicine, Department of Public Health Istanbul University‐Cerrahpaşa İstanbul Türkiye; ^3^ Istanbul Provincial Health Directorate İstanbul Türkiye; ^4^ Güçlükonak State Hospital Şırnak Türkiye; ^5^ Uludere State Hospital Şırnak Türkiye; ^6^ Faculty of Medicine, Department of Public Health Marmara University İstanbul Türkiye

**Keywords:** caregiver burden, home health care, social support

## Abstract

**Aims and Objectives:**

This study aimed to assess caregiver burden and perceived social support among individuals providing care within the scope of home health services and to examine related sociodemographic factors. The relationship between caregiver burden and perceived social support was also explored.

**Methodological Design and Justification:**

A descriptive observational design targeting the entire population of caregivers registered with home health services in Şırnak Province was adopted. This design allowed for a comprehensive assessment of caregiving dynamics in their natural context.

**Ethical Issues and Approval:**

Ethical approval was obtained from Istanbul Medipol University Ethics Committee (Protocol No. 1315, 26/12/2024). Institutional permission was granted by the Şırnak Provincial Health Directorate. Informed verbal consent was obtained from all participants in line with the Declaration of Helsinki.

**Research Methods, Instruments and/or Interventions; Outcome Measures:**

Data were collected through face‐to‐face interviews using a structured questionnaire. Caregiver burden and perceived social support were measured using the Zarit Caregiver Burden Scale (ZCBS) and the Multidimensional Scale of Perceived Social Support (MSPSS). Data analysis included descriptive statistics, non‐parametric tests, and Spearman's correlation.

**Results:**

Among 1022 caregivers, the mean ZCBS score was 41.24 ± 19.79, and the mean MSPSS score was 50.82 ± 17.48. Sociodemographic factors such as age, gender, education, residence, and caregiving characteristics were significantly associated with caregiver burden and perceived social support (*p* < 0.05). A positive correlation was found between caregiver burden and perceived social support (rs = 0.153, *p* < 0.01).

**Study Limitations:**

The descriptive nature limits causal inference. Self‐reported data may introduce bias, and regional factors may affect generalisability.

**Conclusions:**

Caregivers within home health services experience high burden and moderate social support. These findings emphasise the need to enhance home healthcare quality, provide caregiver support programs, and increase access to training and counselling, especially in rural areas.

## 1 Introduction

The global healthcare system is undergoing a marked transformation in response to demographic shifts and socio‐economic changes, a trend also observable in Türkiye. According to data from the World Health Organisation and the United Nations, the global population aged 60 years and over exceeded 1 billion in 2020 and is projected to increase substantially in the coming decades [1, 2]. Similarly, the proportion of the older population in Türkiye has risen steadily in recent years [3]. These demographic changes have contributed to an increased prevalence of chronic diseases and functional dependency, intensifying the need for alternative models of care.

The growing need for elderly and chronically ill individuals to live independently has accelerated the shift from a hospital‐centreed model to home‐ and community‐based healthcare services. Home health services enable patients to receive care in their own living environments, contributing to improved continuity of care, reduced hospitalisation and infection risks, and enhanced quality of life for both patients and caregivers [4, 5].

In Türkiye, home healthcare services were institutionalised nationwide following the enactment of the Directive on the Implementation Procedures and Principles of Home Health Services Provided by the Ministry of Health on May 2, 2010 [6]. These services encompass a wide range of interventions, such as physical examinations, diagnostic and therapeutic procedures, medical care, rehabilitation, and counselling [5, 6]. As of 2024, approximately 445,000 individuals were registered as beneficiaries of home healthcare services in Türkiye, with a cumulative total of 2,780,050 service users since 2010 [7, 8].

Despite the expansion of home health services, caregiving responsibilities often fall on informal caregivers, such as spouses, children, or close relatives, particularly in rural areas where professional support is limited. Prolonged informal caregiving may impose long‐term physical, emotional, social, and economic strain. In the literature, caregiver burden refers to the negative consequences of sustained caregiving, including stress, anxiety, depression, burnout, and social isolation [9, 10]. Moreover, changes in family structure and socio‐economic conditions may further intensify caregiving responsibilities for a limited number of individuals.

Social support is widely recognised as a crucial protective factor that may mitigate the adverse effects of caregiving. Emotional, material, and informational support from family members, friends, and formal social networks has been shown to enhance caregivers' coping capacity and psychological resilience, thereby improving their quality of life [11, 12].

The present study is grounded in Pearlin et al.'s (1990) Stress Process Model, which conceptualises caregiving as a dynamic stress process in which background contextual factors, including sociodemographic characteristics and socioeconomic conditions, shape primary caregiving stressors, while mediating resources such as social support influence caregiver well‐being outcomes. This framework provides a theoretically coherent basis for examining the interplay between caregiver burden, perceived social support, and sociodemographic determinants in a rural home health context [13]. In this context, examining caregiver burden and perceived social support is essential for improving caregivers' quality of life and enhancing the effectiveness of home health services. Therefore, this study aimed to assess caregiver burden and perceived social support among individuals providing care within the scope of home health services, to explore the relationship between these variables, and to examine differences according to caregivers' sociodemographic characteristics.

## 2 Materials and Methods

### 2.1 Study Design and Sample

This study employed a descriptive, observational research design. The target population comprised family members or relatives providing care to patients enrolled in the home health care program in Şırnak Province, Türkiye. The study was conducted through home health units operating under the Şırnak Provincial Health Directorate, including Şırnak State Hospital, CizreSelahattinCizrelioglu State Hospital, Silopi State Hospital, İdil State Hospital, Uludere State Hospital, Beytüşşebap State Hospital, and Güçlükonak State Hospital.

Instead of applying a sampling method, the study aimed to reach all caregivers of 1301 patients receiving regular monthly follow‐up visits. Of the 1301 caregivers contacted, 268 declined participation. After excluding 11 incomplete or erroneous responses, data from 1022 participants were included in the final analysis. Data collection took place between February 1 and March 31, 2025.

Each home care team consists of physicians, nurses, and other healthcare professionals, providing regular monthly visits to patients. When necessary, multidisciplinary support including psychologists, physiotherapists, and social workers is incorporated. During these visits, data collection was carried out through face‐to‐face administration of a structured questionnaire to caregivers. Two preparatory meetings were held with home health care staff to ensure standardisation of the survey implementation. Potential language barriers were addressed with the support of local health personnel acting as interpreters. Survey questions were presented in a comprehensible manner, with clarification sought when needed through family members.

### 2.2 Data Collection Instruments

Data were collected using a five‐part questionnaire delivered via Google Forms. The first two sections captured caregivers' sociodemographic characteristics and caregiving status, based on existing literature. The third section included sociodemographic information of the care recipients.

The fourth section employed the Zarit Caregiver Burden Scale (ZCBS), originally developed by Zarit et al. and adapted into Turkish by İnci and Erdem (2006) [14]. The ZCBS consists of 22 items rated on a 5‐point Likert scale (0 = Never to 4 = Nearly always), with total scores ranging from 0 to 88. Higher scores indicate a higher caregiving burden. Burden levels were categorised according to established cut‐off scores: 0–20 indicates no or little caregiving burden, 21–40 moderate caregiving burden, 41–60 severe caregiving burden, and 61–88 extreme caregiving burden. The scale's internal consistency was previously reported as 0.95; in the present study, Cronbach's α was 0.953, indicating excellent reliability within this sample.

The fifth section used the Multidimensional Scale of Perceived Social Support (MSPSS), developed by Zimet et al. and validated in Turkish by Eker et al. [15]. The MSPSS includes 12 items across three subscales: family, friends, and significant others. Each item is rated on a 7‐point Likert scale. Total scores range from 12 to 84, with higher scores reflecting greater perceived social support. In the present study, the internal consistency of the MSPSS was excellent overall (Cronbach's α = 0.933), with subscale coefficients of 0.889 for the family support subscale, 0.924 for the friend support subscale, and 0.908 for the significant other support subscale.

### 2.3 Data Analysis

Data were analysed using SPSS version 25.0. Continuous variables were presented as mean ± standard deviation (SD) and median (min–max), while categorical variables were summarised as frequencies and percentages.

Normality was assessed using the Kolmogorov–Smirnov and Shapiro–Wilk tests, alongside skewness and kurtosis values (accepted range±1.5). Chi‐square and Fisher's exact tests were used for categorical comparisons. Non‐parametric tests (Mann–Whitney U and Kruskal‐Wallis) were applied to continuous variables due to the non‐normal distribution of data. Spearman's rank correlation was used to evaluate relationships between continuous variables. A *p*‐value of < 0.05 was considered statistically significant.

### 2.4 Ethical Considerations

The study received approval from the Ethics Committee of Istanbul Medipol University (Protocol No. 1315, dated 26/12/2024). Institutional permission was obtained from the Şırnak Provincial Health Directorate (Decision No. 280529882, dated 20/01/2025). Participants were fully informed about the study objectives, and verbal informed consent was obtained. The research adhered to the principles outlined in the Declaration of Helsinki.

## 3 Results

The mean age of the caregivers was 41.71 ± 12.03 years. Most participants were female (80.2%), had primary school education or less (71.9%), resided in villages, towns, or districts (90.0%), and reported having an income lower than their expenses (58.6%). Additionally, 70.2% of caregivers had three or more children, 80.8% lived in households of five or more people, and 56.9% were first‐degree relatives of the patient. The detailed sociodemographic characteristics of the caregivers are presented in Table 1.

**TABLE 1:** Sociodemographic characteristics of caregivers.

	Mean ± SD	Median (Min‐Max)
	Number (*n*)	Percent (%)
**Age**	41.71 ± 12.03	41 (15–89)
**Age Group**		
15–30 Years	180	17.6
31–44 Years	448	43.8
45–59 Years	324	31.7
≥ 60 Years	70	6.8
**Gender**		
Female	820	80.2
Male	202	19.8
**Marital Status**		
Single	184	18.0
Married	804	78.7
Widowed/Divorced	34	3.3
**Educational Level**		
Illiterate	363	35.5
Literate	200	19.6
Primary School	172	16.8
Secondary School	95	9.3
High School	133	13.0
University	59	5.8
**Residence**		
Village/Town	312	30.5
District	608	59.5
City	102	10.0
**Occupational Status**		
Working	145	14.2
Not Working	877	85.8
**Social Security**		
Yes	734	71.8
No	288	28.2
**Economic Situation**		
Income Less than Expenditure	599	58.6
Income Equivalent to Expenditure	409	40.0
Income More than Expenditure	14	1.4
**Number of Children**	4.03 ± 2.87	4 (0–17)
0	205	20.1
1–2	99	9.7
3–4	271	26.5
5–6	237	23.2
7 and above	210	20.5
**Number of People in the House**	6.90 ± 2.65	7 (1–20)
1–2	42	4.1
3–4	154	15.1
5–6	259	25.3
7 and above	567	55.5
**Continuous Medication Use**		
Yes	267	26.1
No	755	73.9
**Relationship to care recipient**		
Parent	216	21.1
Partner	78	7.6
Children	247	24.2
Brother/Sister	41	4.0
Daughter‐in‐law/Son‐in‐law	393	38.5
Others (relative/neighbour/acquaintance)	47	4.6
**Total**	**1022**	**100**

Abbreviations: Max: Maximum, Min: Minimum, SD: Standard Deviation.

Regarding caregiving status, 92.4% of caregivers provided care due to family obligations, 48.3% had been providing care for more than 5 years, and 95.7% provided care daily. Furthermore, 88.8% reported encountering difficulties in patient hygiene care, and 20.0% expressed a need for psychological support during the caregiving process. The majority (77.7%) of participants rated the home health care services positively. Details on caregiving status are provided in Table 2.

**TABLE 2:** Caregiving situation.

	Number (*n*)	Percent (%)
**Reason for Providing Care to the Patient**		
Family Responsibility	944	92.4
No One Else to Provide Care	248	24.3
Economic Reasons	15	1.5
Other	10	1.0
**Duration of Providing Care, Help and Support**	88.35 ± 78.11	60 (0.5–720)
Less than 1 Year	83	8.1
1–3 Years	196	19.2
3–5 Years	249	24.4
6–10 Years	305	29.8
Above 10 Years	189	18.5
**Frequency of Patient Care Provision**		
Everyday	978	95.7
4–5 days a week	34	3.3
1–2 days a week or less	10	1.0
**Average, daily care hours given**	6.48 ± 5.70	5 (0.33–24)
Less than 1 h	109	10.7
1–3 h	226	22.1
4–6 h	371	36.3
7–10 h	152	14.9
More than 10 h	164	16.0
**Receiving Carer's Salary**		
Yes	559	54.7
No	463	45.3
**Receiving Patient Care Training Status**		
Yes	446	43.6
No	576	56.4
**Feeling of Inadequacy in Caregiving**		
Yes	472	46.2
No	550	53.8
**Difficulty in Providing Care**		
Cleaning of the patient	908	88.8
Nutrition of the patient	552	54.0
Medication of the patient	298	29.2
Use of the device/instrument requiring care	104	10.2
Other	60	5.9
**Existence of Other Dependents at Home**		
Yes	412	40.3
No	610	59.7
**Experiencing Physical Discomfort Due to Caregiving**		
Yes	552	54.0
No	470	46.0
**Experiencing Psychological Discomfort Due to Caregiving**		
Yes	259	25.3
No	763	74.7
**Desire to Receive Psychological Support**		
Yes	204	20.0
No	818	80.0
**Level of Contribution of Home Health Services**		
Low	12	1.2
Medium	216	21.1
High	794	77.7
**Total**	**1022**	**100**

With respect to patient characteristics, 73.9% of the cared‐for patients were aged over 60 years, 95.8% had primary school education or less, 63.9% were on four or more medications daily, 54.6% were fully dependent on others for physical care, and 47.7% had special needs. These data are summarised in Table 3.

**TABLE 3:** Characteristics of patients.

	Mean ± SD	Median (Min‐Max)
	Number (*n*)	Percent (%)
**Age**	65.8 ± 24.9	74 (3–125)
**Age Group**		
< 18 Age	91	8.9
18–30 Age	66	6.5
31–44 Age	33	3.2
45–59 Age	77	7.5
≥ 60 Age	755	73.9
**Gender**		
Female	589	57.6
Male	433	42.4
**Educational Level**		
Illiterate	827	80.9
Literate	101	9.9
Primary School	51	5.0
Secondary School	21	2.1
High School	20	2.0
University	2	0.2
**Social Security**		
Yes	805	78.8
No	217	21.2
**Chronic Disease**		
Cardiovascular Diseases	614	60.1
Neurological Diseases	468	45.8
Endocrine Diseases	359	35.1
Respiratory System Diseases (COPD‐Asthma)	96	9.4
Cancer	47	4.6
Other	316	30.9
**Number of medications used**	**4.27 ± 2.25**	**4 (0–13)**
**Polypharmacy (≥ 4 medications)**		
Yes	653	63.9
No	369	36.1
**Disability certificate**		
Yes	746	73.0
No	276	27.0
**Physical Dependency Status**		
Fully Dependent	558	54.6
Partially Dependent	411	40.2
Independent	53	5.2
**Nutritional Status**		
Oral	867	84.8
Special Formula	161	15.8
N/G Tube	65	6.4
Gastrostomy	23	2.3
Other	18	1.7
**Urinary or Nasogastric Tube Status**		
Yes	743	72.7
No	279	27.3
**Tracheostomy Status**		
Yes	44	4.3
No	978	95.7
**Bed Sore Status**		
Yes	250	24.5
No	772	75.5
**Chemotherapy/Radiotherapy Status**		
Yes	32	3.1
No	990	96.9
**Patient's Special Needs (Injection, dressing, physical therapy) Status**		
Yes	487	47.7
No	535	52.3
**Total**	**1022**	**100**

Abbreviations: Max: Maximum, Min: Minimum, SD: Standard Deviation.

The mean Zarit Caregiver Burden Scale score and the categorical distribution of burden levels are shown in Table 4. The scores of the Multidimensional Scale of Perceived Social Support and its subscales are presented in Table 5.

**TABLE 4:** Caregiver Burden Scale Scores.

	Mean ± SD	Median (Min‐Max)
	Number (*n*)	Percent (%)
**ZCBS**	41.24 ± 19.79	38 (0–88)
**ZCBS Subscale**		
No or Little Caregiving Burden	126	12.3
Moderate Caregiving Burden	435	42.6
Severe Caregiving Burden	279	27.3
Extreme Caregiving Burden	182	17.8
**Total**	**1022**	**100**

Abbreviations: Max: Maximum, Min: Minimum, SD: Standard Deviation, ZCBS: Zarit Caregiver Burden Scale.

**TABLE 5:** Multidimensional Perceived Social Support Scale and subscale scores.

	Mean ± SD	Median (Min‐Max)
**MSPSS Total Score**	50.82 ± 17.48	51 (12–84)
**MSPSS Subscale Score**		
Perceived Family Support	19.75 ± 6.08	20 (4–28)
Perceived Friend Support	15.91 ± 7.18	16 (4–28)
Perceived Significant Other Support	15.16 ± 7.18	15 (4–28)
**Total**	**1022**	**100**

Abbreviations: Max, Maximum; Min, Minimum; MSPSS, Multidimensional Perceived Social Support Scale; SD, Standard Deviation.

The caregiver burden was found to be significantly higher among individuals aged 45–49, married and widowed/divorced participants, literate individuals compared to those with no formal education, primary school graduates compared to high school graduates, and residents of villages or towns compared to those in districts or cities (*p* < 0.05). Caregivers with three to four children, those providing care for less than one hour per day, those reporting inadequacy in their caregiving role, caregivers with additional caregiving responsibilities at home, and those experiencing physical or psychological discomfort due to caregiving reported higher burden levels (*p* < 0.05). Detailed findings on caregiver burden are provided in Table 6.

**TABLE 6:** The burden of caregiving according to the sociodemographic and other characteristics of caregivers.

	Number (*n*)	Rank Mean	Test Statistic	*p*
**Age Group**				
15–30 Years	180	453.28^1^	KWH 9.978	0.019^1–3^
31–44 Years	448	511.84^2^		
45–59 Years	324	543.96^3^		
≥ 60 Years	70	496.06^4^		
**Gender**				
Female	820	512.93	MWU 81.646	0.755
Male	202	505.69		
**Marital Status**				
Single	184	465.68^1^	KWH 6.784	0.034^1–2,1–3^
Married	804	519.10^2^		
Widowed/Divorced	34	576.66^3^		
**Educational Level**				
Illiterate	363	494.55^1^	KWH 48.136	< 0.001^2–1,3,4,5,6,3–5^
Literate	200	620.67^2^		
Primary School	172	527.09^3^		
Secondary School	95	502.06^4^		
High School	133	408.24^5^		
University	59	448.28^6^		
**Residence**				
Village/Town	312	579.44^1^	KWH 88.828	< 0.001^1–2,1–3,2–3^
District	608	518.22^2^		
City	102	263.61^3^		
**Occupational Status**				
Working	145	511.63	MWU 63.467	0.972
Not Working	877	510.71		
**Social Security**				
Yes	734	498.41	MWU 96.089	0.024
No	288	544.86		
**Economic Situation**				
Income Less than Expenditure	599	519.47	KWH 1.504	0.471
Income Equivalent to Expenditure	409	498.44		
Income More than Expenditure	14	552.18		
**Number of Children**				
0	205	462.61^1^	KWH 16.011	0.003^1–3,2–3^
1–2	99	441.77^2^		
3–4	271	541.82^3^		
5–6	237	533.85^4^		
7 and above	210	527.75^5^		
**Number of People in the House**				
1–2	42	489.00	KWH 6.284	0.099
3–4	154	498.94		
5–6	259	551.08		
7 and above	567	498.50		
**Continuous Medication Use**				
Yes	267	505.81	MWU 99.274	0.714
No	755	513.51		
**Relationship to care recipient**				
Parent	216	572.53^1^	KWH 21.556	0.001^1–4,1–6^
Partner	78	512.44^2^		
Children	247	494.07^3^		
Brother/Sister	41	382.17^4^		
Daughter‐in‐law/Son‐in‐law	393	511.96^5^		
Others (relative/neighbour/acquaintance)	47	430.06^6^		
**Duration of Providing Care, Help and Support**				
Less than 1 Year	83	476.89	KWH 4.688	0.321
1–3 Years	196	536.68		
3–5 Years	249	500.94		
6–10 Years	305	526.64		
Above 10 Years	189	490.06		
**Frequency of Patient Care Provision**				
Everyday	978	508.60	KWH 1.765	0.414
4–5 days a week	34	574.16		
1–2 days a week or less	10	472.06		
**Average, daily care hours given**				
Less than 1 h	109	564.17^1^	KWH 47.610	< 0.001^1–5,2–3,2–4,2–5^
1–3 h	226	613.59^2^		
4–6 h	371	476.32^3^		
7–10 h	152	490.44^4^		
More than 10 h	164	434.93^5^		
**Receiving Carer's Salary**				
Yes	559	538.98	MWU 114.046	0.001
No	463	478.32		
**Receiving Patient Care Training Status**				
Yes	446	536.04	MWU 117.501	0.019
No	576	492.49		
**Feeling of Inadequacy in Caregiving**				
Yes	472	645.21	MWU 66.689	< 0.001
No	550	396.75		
**Existence of Other Dependents at Home**				
Yes	412	604.23	MWU 87.457	< 0.001
No	610	448.87		
**Experiencing Physical Discomfort Due to Caregiving**				
Yes	552	575.73	MWU 94.263	< 0.001
No	470	436.06		
**Experiencing Psychological Discomfort Due to Caregiving**				
Yes	259	713.51	MWU 46.487	< 0.001
No	763	442.93		
**Desire to Receive Psychological Support**				
Yes	204	760.86	MWU 32.567	< 0.001
No	818	449.31		
**Level of Contribution of Home Health Services**				
Low	12	494.54	KWH 5.814	0.055
Medium	216	468.92		
High	794	523.34		

*Note:* ^1,2^:Refers to paired groups where there is a significant statistical difference, 1–6: It refers to group numbers.

Abbreviations: KWH: Kruskal‐Wallis H Test, MWU: Mann Whitney U test, ZCBS: Zarit Caregiver Burden Scale.

Significant differences in perceived social support scores were observed based on gender, marital status, education level, and place of residence (*p* < 0.05). Notably, caregivers who had provided care for over 10 years reported higher perceived family support scores. Those providing less than ten hours of daily care had higher perceived family support, while those providing less than three hours reported higher perceived support from friends and significant others. Additionally, caregivers responsible for another person at home and those who rated home health care services highly also showed higher perceived social support scores (*p* < 0.05). These comparisons are outlined in Table 7.

**TABLE 7:** MSPSS scale scores according to caregivers' sociodemographic and other characteristics.

		MSPSS			
		Perceived Family Support	Perceived Friend Support	Perceived Significant Other Support	MSPSS
	Number (*n*)	Rank Mean	Rank Mean	Rank Mean	Rank Mean
**Age Group**					
15–30 Years	180	574.39^1^	562.04^1^	518.77	557.01
31–44 Years	448	488.24^2^	488.56^2^	502.51	489.86
45–59 Years	324	504.48^3^	506.79^3^	512.18	508.84
≥ 60 Years	70	531.15^4^	550.14^4^	547.2	545.28
**Test Statistic (KWH)**		11.522	9.288	1.555	7.634
*p*		0.009^1–2^	0.026^1–2^	0.67	0.054
**Gender**					
Female	820	502.76	499.37	504.16	499.21
Male	202	547.00	560.75	541.3	561.39
**Test Statistic (KWH)**		75.650	72.870	76.8	72.741
*p*		0.056	0.008	0.109	0.007
**Marital Status**					
Single	184	561.35^1^	521.47	492.86	524.1
Married	804	507.87^2^	511.42	517.89	512.94
Widowed/Divorced	34	327.65^3^	459.49	461.13	409.19
**Test Statistic (KWH)**		18.685	1.269	2.107	4.441
*p*		< 0.001^1–3,2–3^	0.530	0.349	0.109
**Educational Level**					
Illiterate	363	474.20^1^	460.98^1^	476.541	457.621
Literate	200	448.29^2^	572.27^2^	587.352	556.022
Primary School	172	559.84^3^	480.25^3^	505.743	513.853
Secondary School	95	548.82^4^	510.94^4^	491.164	518.514
High School	133	572.11^5^	533.92^5^	504.655	538.475
University	59	617.63^6^	657.77^6^	534.456	613.176
**Test Statistic (KWH)**		34.567	36.388	19.297	24.834
*p*		< 0.001^1–3,5,6,2–3,5,6^	< 0.001^1–2,6,3–2,6,4–6^	0.002^1–2^	< 0,001^1–2,6^
**Residence**					
Village/Town	312	568.58^1^	459.82^1^	473.431	493.081
District	608	450.18^2^	495.35^2^	490.592	477.282
City	102	702.43^3^	765.84^3^	752.573	771.823
**Test Statistic (KWH)**		81.128	87.339	76.486	88.768
*p*		< 0.001^1–2,3,2–3^	< 0.001^1–3,2–3^	< 0.001^1–3,2–3^	< 0,001^1–3,2–3^
**Occupational Status**					
Working	145	510.20	499.83	505.73	503.13
Not Working	877	519.36	582.07	546.4	562.13
**Test Statistic (MWU)**		62.443	53.350	58.521	56.241
*p*		0.728	0.002	0.124	0.026
**Social Security**					
Yes	734	508.27	518.05	511.01	514.23
No	288	519.72	494.81	512.74	504.54
**Test Statistic (MWU)**		103.328	100.889	105.338	103.692
*p*		0.576	0.257	0.933	0.637
**Economic Situation**					
Income Less than Expenditure	599	527.40	501.89	513.77	512.16
Income Equivalent to Expenditure	409	488.92	521.34	506.25	507.76
Income More than Expenditure	14	490.86	635.39	567.71	592.54
**Test Statistic (KWH)**		4.229	3.565	0.674	1.125
*p*		0.121	0.168	0.714	0.57
**Number of Children**					
0	205	551.77^1^	528.88^1^	499.951	525.341
1–2	99	587.07^2^	621.09^2^	609.102	619.482
3–4	271	468.65^3^	515.71^3^	521.913	505.563
5–6	237	474.62^4^	509.70^4^	518.764	503.034
7 and above	210	533.49^5^	439.47^5^	455.145	464.305
**Test Statistic (KWH)**		21.022	26.994	19.328	19.383
*p*		< 0.001^1–3,2–3,4^	< 0.001^1–5,2–3,4,5,3–5^	0.001^1–2,2–5^	0.001^2–3,4,5^
**Number of People in the House**					
1–2	42	480.31	510.07^1^	499.331	492.351
3–4	154	565.62	584.18^2^	579.782	585.502
5–6	259	510.20	539.01^3^	531.963	536.25
7 and above	567	499.70	479.30^4^	484.514	481.524
**Test Statistic (KWH)**		6.601	18.379	14.337	17.534
*p*		0.086	< 0.001^2–4,3–4^	0.002^2–4^	0.001^2–4^
**Continuous Medication Use**					
Yes	267	563.70	520.06	519.83	534.78
No	755	493.04	508.47	508.56	503.27
**Test Statistic (MWU)**		86.854	98.506	98.569	94.575
*p*		0.001	0.581	0.591	0.134
**Relationship to care recipient**					
Parent	216	552.57^1^	542.46^1^	535.461	551.031
Partner	78	521.33^2^	535.27^2^	575.002	543.762
Children	247	509.86^3^	521.40^3^	487.473	505.923
Brother/Sister	41	499.62^4^	523.34^4^	587.954	540.824
Daughter‐in‐law/Son‐in‐law	393	472.20^5^	472.63^5^	488.845	472.805
Others (relative/neighbour/acquaintance)	47	654.03^6^	592.48^6^	545.116	603.666
**Test Statistic (KWH)**		22.416	13.614	12.380	16.643
*p*		< 0.001^1–5,2–6 3–6,4–6,5–6^	0.018^1–5,5–6^	0.030^2–3,2–5,3–4,4–5^	0.005^1–5,5–6^
**Duration of Providing Care, Help and Support**					
Less than 1 Year	83	593.11^1^	547.34^1^	543.461	568.471
1–3 Years	196	492.80^2^	519.30^2^	544.432	522.482
3–5 Years	249	466.04^3^	505.74^3^	500.223	485.623
6–10 Years	305	481.90^4^	498.81^4^	480.044	485.534
Above 10 Years	189	602.71^5^	515.74^5^	528.965	551.105
**Test Statistic (KWH)**		34.383	2.063	7.924	11.046
*p*		< 0.001^1–3,4,2–5,3–5,4–5^	0.724	0.094	0.026^1–3,4,3–5,4–5^
**Frequency of Patient Care Provision**					
Everyday	978	511.29	506.09^1^	507.141	507.13
4–5 days a week	34	504.22	616.76^2^	643.442	613.97
1–2 days a week or less	10	557.05	682.90^3^	489.003	590.45
**Test Statistic (KWH)**		1.261	8.045	7.084	5.030
*p*		0.878	0.018^1–2^	0.029^1–2^	0.081
**Average, daily care hours given**					
Less than 1 h	109	536.33^1^	649.69^1^	637.501	631.821
1–3 h	226	552.13^2^	574.35^2^	582.632	582.232
4–6 h	371	542.57^3^	470.94^3^	481.603	491.003
7–10 h	152	497.08^4^	464.42^4^	440.454	456.004
More than 10 h	164	382.07^5^	468.43^5^	463.225	431.875
**Test Statistic (KWH)**		41.338	48.625	50.124	50.211
*p*		< 0.001^1–5,2–5,3–5,4–5^	< 0.001^5–1,2,4–1,2,3–1,2^	< 0.001^5–1,2,4–1,2,3–1,2^	< 0.001^5–1,2,4–1,2,3–1,2^
**Receiving Carer's Salary**					
Yes	559	548.77	532.99	544.39	548.62
No	463	466.50	485.56	471.79	466.69
**Test Statistic (MWU)**		108.574	117.397	111.022	108.66
*p*		< 0.001	0.010	< 0.001	< 0.001
**Receiving Patient Care Training Status**					
Yes	446	560.93	598.00	601.18	604.05
No	576	473.22	444.52	442.06	439.83
**Test Statistic (MWU)**		106.400	89.868	88.449	87.168
*p*		< 0.001	< 0.001	< 0.001	< 0.001
**Feeling of Inadequacy in Caregiving**					
Yes	472	461.60	540.65	545.77	525.08
No	550	554.32	486.48	482.09	499.85
**Test Statistic (MWU)**		106.248	116.039	113.623	123.39
*p*		< 0.001	0.003	0.001	0.173
**Existence of Other Dependents at Home**					
Yes	412	523.14	556.15	581.48	563.92
No	610	503.64	481.35	464.23	476.1
**Test Statistic (MWU)**		120.863	107.265	96.827	104.065
*p*		0.298	< 0.001	< 0.001	< 0.001
**Experiencing Physical Discomfort Due to Caregiving**					
Yes	552	498.20	498.34	506.49	500.45
No	470	527.12	526.96	517.38	524.47
**Test Statistic (MWU)**		122.377	122.453	126.955	123.622
*p*		0.117	0.122	0.556	0.195
**Experiencing Psychological Discomfort Due to Caregiving**					
Yes	259	584.36	610.44	632.41	630.77
No	763	486.77	477.91	470.46	471.01
**Test Statistic (MWU)**		79.939	73.183	67.493	67.917
*p*		< 0.001	< 0.001	< 0.001	< 0.001
**Desire to Receive Psychological Support**					
Yes	204	521.76	636.03	645.8	629.91
No	818	508.94	480.44	478.01	481.97
**Test Statistic (MWU)**		81.343	58.032	56.039	59.281
*p*		0.578	< 0.001	< 0.001	< 0.001
**Level of Contribution of Home Health Services**					
Low	12	576.38^1^	347.42^1^	334.631	390.631
Medium	216	427.41^2^	416.73^2^	405.742	404.142
High	794	533.40^3^	539.76^3^	542.943	542.533
**Test Statistic (KWH)**		22.631	33.339	41.161	39.384
*p*		< 0.001^2–3^	< 0.001^2–3^	< 0.001^1–3,2–3^	< 0.001^2–3^

*Note:* ^1,2^:Refers to paired groups where there is a significant statistical difference. 1–6 It refers to group numbers.

Abbreviations: KWH: Kruskal‐Wallis H Test, MWU: Mann Whitney U test, MSPSS: Multidimensional Perceived Social Support Scale.

A statistically significant positive correlation was found between the Zarit Caregiver Burden Scale and the Multidimensional Scale of Perceived Social Support (rs = 0.153, *p* < 0.01), indicating that higher perceived social support was associated with higher reported caregiving burden. The correlation analysis is presented in Table 8.

**TABLE 8:** Correlation between certain characteristics of caregivers and Zarit Caregiver Burden Scale scores and Multidimensional Perceived Social Support Scale scores (*n* = 1022).

	ZCBS	MSPSS	Perceived Friend Support	Perceived Family Support	Perceived Significant Other Support	Age of Caregiver	Duration of care provision
**ZCBS** (rs)	1.000						
**MSPSS** (rs)	0.153**	1.000					
**Perceived Friend Support** (rs)	0.027	0.705*	1.000				
**Perceived Family Support** (rs)	0.098**	0.905**	0.449**	1.000			
**Perceived Significant Other Support** (rs)	0.196**	0.913**	0.808**	0.467**	1.000		
**Age of Caregiver** (rs)	0.078*	0.002	−0.008	−0.019	0.030	1.000	
**Duration of care provision** (rs)	−0.008	−0.003	−0.023	0.056	−0.034	0.247**	1.000

*Note:* *: *p* < 0.05, **: *p* < 0.01.

Abbreviations: MSPSS: Multidimensional Perceived Social Support Scale, rs: Spearman Correlation, ZCBS: Zarit Caregiver Burden Scale.

## 4 Discussion

The present study aimed to assess the levels of caregiver burden and perceived social support among caregivers of patients receiving home health services. Interpreted through the Stress Process Model, these findings suggest that structural disadvantages characteristic of the study setting, including rural residence, low educational attainment, and economic hardship, function as background contextual factors that amplify primary caregiving stressors, while perceived social support appears to operate as an insufficient mediating resource in this population [13]. The findings revealed that 87.7% of participants experienced a moderate to high level of caregiving burden, a notably higher rate than that reported in comparable studies [12, 16, 17]. Several region‐specific factors in Şırnak were likely associated with this outcome, including rural living conditions, socio‐economic disadvantages, and low educational attainment.

Consistent with prevailing gender norms, caregiving roles are often assumed by women. International studies, such as those by Hong et al. in Korea, Perez et al. in Spain, and Pacheco Barzallo et al. in Switzerland, have consistently reported that approximately two‐thirds of family caregivers are female [18, 19, 20]. Similarly, Turkish studies confirm that caregiving is predominantly undertaken by married women [16, 17, 21, 22, 23]. Notably, caregivers aged 45–49 years reported significantly higher burden levels than younger caregivers. This finding may reflect the cumulative physical, emotional, and socio‐economic pressures associated with midlife, when caregiving responsibilities often coexist with employment, childcare, and household duties [24]. The presence of additional dependents within crowded households further amplified caregiver burden.

Educational level and place of residence emerged as important determinants of caregiver burden. In line with previous research, lower educational attainment was associated with higher perceived burden [25, 26, 27]. Limited education may be linked to lower health literacy, reduced access to care‐related information, and less effective coping strategies, which may in turn relate to higher caregiving strain. Place of residence was another significant factor, with caregivers living in villages and towns reporting higher burden than those residing in district or city centres. Rural caregivers often face restricted access to healthcare services, transportation difficulties, and limited social and professional support. These structural disadvantages, combined with economic hardship and unemployment, may be associated with greater physical and psychological strain and a higher likelihood of caregiver burnout [16, 22, 23, 26, 28, 29, 30].

Contrary to common assumptions in the literature, this study found that caregivers who had provided care for a shorter duration reported higher levels of burden. While many studies suggest a linear increase in burden with prolonged caregiving, similar non‐linear patterns have also been reported [31, 32]. This finding indicates that caregiving burden may be influenced more by care complexity, preparedness, and psychological adaptation than by duration alone. The perception of caregiving as a familial obligation, reported by most participants, as well as limited formal training, may be associated with this pattern. Additionally, the subjective measurement of caregiving duration may have influenced caregivers' perceptions of burden [23, 30, 33].

In this study, caregivers reported a moderate level of perceived social support, with family identified as the primary source of support, followed by friends and significant others. This pattern is consistent with findings from rural and semi‐rural settings, where traditional family structures play a central role in caregiving processes [10, 34, 35, 36]. Lower levels of support from friends and significant others may be explained by caregivers' sociodemographic characteristics, including gender, marital status, limited education, and restricted social participation, as well as crowded living conditions and limited social networks [34, 35, 36, 37, 38]. In addition, caregivers who perceived the contribution of home health services as high also reported higher levels of perceived support from significant others. This finding suggests that positive evaluations of professional home health services may be associated with caregivers' broader perceptions of social support. Professional home health services may be associated with better psychosocial outcomes and lower levels of perceived loneliness among caregivers.

A statistically significant positive association was observed between perceived social support and caregiver burden. While social support is often considered a protective factor, this finding suggests that higher perceived support may coexist with higher burden in certain caregiving contexts. Caregivers experiencing greater burden may be more likely to seek, recognise, or report social support as a coping response. Indeed, the study conducted by Aksin et al. in 2023 revealed that elevated levels of care burden were associated with an increased propensity to seek support and to engage in social participation [39]. Alternatively, perceived social support may not fully correspond to caregivers' actual needs or may be linked to additional expectations and responsibilities. Given the descriptive nature of the study, the direction and mechanisms of this relationship cannot be determined. These findings underscore the complex and multidimensional nature of caregiving experiences.

This study offers valuable insight into the caregiving experiences of individuals in a socioeconomically disadvantaged and rural region of Türkiye, supported by a large and inclusive sample that enhances its representativeness of the findings within the study setting. The use of validated measurement tools and face‐to‐face data collection may have strengthened data reliability. However, several limitations should be considered. The descriptive design limits the ability to draw causal inferences regarding the relationships between caregiver burden, perceived social support, and associated factors. Data were based on self‐reported measures, which may be subject to recall bias and social desirability bias. In addition, caregiving characteristics such as duration and intensity were assessed according to caregivers' perceptions rather than objective indicators. Furthermore, multiple comparisons were conducted without adjustment for Type I error, which may increase the risk of spurious findings and should be interpreted with caution. Finally, the region‐specific context and the rural and socio‐economic characteristics of the study population may limit the generalisability of the findings to other regions or healthcare settings.

## 5 Conclusion

This study demonstrated that most caregivers providing care within the scope of home health services experienced a high level of caregiving burden, while their perceived social support remained at a moderate level. The findings revealed a significant association between caregiver burden and perceived social support, with various sociodemographic factors such as age, gender, education, place of residence, and caregiving circumstances influencing both variables.

Importantly, the study underscores that beyond the mere presence of social support, its functionality and perceived adequacy play a crucial role in shaping the caregiving experience. Moreover, the observation that caregivers who perceived greater benefits from home health services also reported higher levels of perceived support from significant others suggests that professional care services may indirectly strengthen caregivers' social support networks.

Considering these findings, it is essential to enhance the quality and accessibility of home health services, provide targeted psychosocial support for caregivers, promote health literacy, and expand training and counselling services particularly for caregivers in rural settings. Additionally, the development of comprehensive intervention programs that address both the availability and the effectiveness of social support systems is critical to alleviating caregiver burden and improving overall care outcomes.

## Author Contributions

Conceptualisation: F.O., M.S.D., K.T.A. and H.A. Methodology: F.O., M.S.D., K.T.A. and Z.M.A. Formal analysis: F.O., M.S.D. and K.T.A. Investigation: F.O., M.S.D., K.T.A., H.A., A.I., B.B., K.A., E.A., D.G. and Z.M.A. Data curation: F.O., M.S.D., K.T.A. and H.A. Writing – original draft: F.O., M.S.D. and K.T.A. Writing – review and editing: F.O., M.S.D. and K.T.A. Supervision: F.O., M.S.D. and K.T.A.

## Funding

The authors have nothing to report.

## Conflicts of Interest

The authors declare no conflicts of interest.

## Data Availability

The data that support the findings of this study are available from the corresponding author upon reasonable request.
